# Editorial: Neurologic Correlates of Motor Function in Cerebral Palsy: Opportunities for Targeted Treatment

**DOI:** 10.3389/fnhum.2020.615397

**Published:** 2020-11-19

**Authors:** Jessica Rose, Christos Papadelis, Deborah Gaebler-Spira

**Affiliations:** ^1^Division of Pediatric Orthopaedics, Stanford University School of Medicine, Stanford, CA, United States; ^2^Jane and John Justin Neurosciences Center, Cook Children's Health Care System, Fort Worth, TX, United States; ^3^Shirley Ryan AbilityLab, Chicago, IL, United States

**Keywords:** cerebral palsy, neurology, mobility, gait, upper limb function

Cerebral palsy (CP) is the most common childhood motor disorder. It is a clinical syndrome with several etiologies and associated symptoms. These symptoms or impairments lead to varying degrees of gross and fine motor severity and impact functional activities such as gait, arm use, and speech. Current medical and surgical treatments for CP are only partially effective in improving these motor abnormalities and may cause significant muscle weakness and other complications. Thus, for persons with CP meaningful improvements remain elusive. We believe that a better understanding of the mechanisms underlying neuromuscular deficits of CP will lead to more effective treatments.

Abnormal tone interfering with the child's posture and development is a common feature of spastic, dyskinetic, and ataxic CP (Sanger et al., 2003, 2006, 2010), which arise from early injury to specific brain regions and result in characteristic neuromuscular deficits. Children with spastic CP, the most common form, have neuromuscular deficits associated with injury to the corticospinal motor track. Subsequent loss of descending neural activation and inhibition result in weak and short muscles that fail to grow sufficiently relative to skeletal growth, and affected muscles have increased sensitivity to stretch (Zhou et al., 2017). Additionally, voluntary movement is limited by impaired selective motor control (SMC), characterized by flexion and extension synergy patterns (Cahill-Rowley and Rose, 2014). Ironically, most research and medical interventions for spastic CP focus on tone, however, function is often most effected by muscle weakness and slow muscle growth relative to bone growth, in combination with impaired SMC. Muscle is frequently overlooked, and as the end organ, it is an area that needs focused attention and clinical research. Current treatments for joint contracture, such as surgical tendon lengthening and skeletal realignment address deformities that are years in the making and require hospitalization. Addressing the underlying impairments of muscle weakness and slow growth rate could prevent joint contracture and skeletal malalignment, and minimize the need for surgery. Muscle is highly responsive to input and substantially influences function, it is therefore a promising target for effective treatment. Understanding the neurological correlates of these neuromuscular impairments can inform more targeted and successful treatment.

Typically, CP is categorized by tone and topography. Many children with CP may have a mixed tone disorder that requires careful attention to identify. Dyskinetic CP is thought to arise primarily from injury to the basal ganglia, causing uncontrolled movements that impose on voluntary movements (Zhou et al., 2017). Ataxic CP is thought to arise primarily from injury to the cerebellum, causing impaired postural balance and targeting (Zhou et al., 2017).

This research article collection, “*Neurologic correlates of motor function in cerebral palsy: opportunities for targeted treatment*” highlights current research from around the world that investigates important structure-function relationships underlying the neuromuscular deficits of CP. This exploratory research can inform and potentially translate into effective treatment.

Here we examine the links between regional brain injury, metabolic activity and motor impairments of the upper (Hung et al.; Papadelis et al.,) and lower limbs (Cahill-Rowley et al.; Fowler et al.; Papageorgiou et al.; Short et al.). In addition, the important role of sensorimotor (Zarkou et al.) and visuospatial (Maioli et al.) impairments in movement abnormalities are investigated. To guide effective treatment, it is vital to delineate motor control deficits from musculoskeletal limitations of gait abnormalities, this is explored using a physics-based simulation (Falisse et al.). An initial investigation of neuromuscular reflex characteristics to predict treatment outcome offers a step toward delivering more precise medicine for persons with CP (Bar-On et al.). Finally, the effects of treatment that targets underlying neuromuscular deficits of CP is examined (Beani et al.; Peeters et al.), including multichannel neuromuscular electrical stimulation that highlights need for lightweight wearable gait devices (Mooney and Rose). We envisioned that this research article collection would provide a context for developing an effective approach to addressing the etiology, diagnosis and treatment of CP. To this end, we appreciate your interest and hope that you will be inspired toward future research.

## Author Contributions

All authors listed have made a substantial, direct and intellectual contribution to the work, and approved it for publication.

## Conflict of Interest

The authors declare that the research was conducted in the absence of any commercial or financial relationships that could be construed as a potential conflict of interest.
